# Supplementary material for: Vitamin D Modified DSS-Induced Colitis in Mice via STING Signaling Pathway
Source: Biology (Basel). 2025 Jun 18;14(6):715. doi: 10.3390/biology14060715 (PMC12190092; doi:10.3390/biology14060715)
Supplement: Supplementary file 1 [file biology-14-00715-s001.zip › Supplementary Materials S1: 16s-report of gut microbiota/Groups/diff_analysis/metagenomeRCAZY/figure/boxplot/top10log.pdf]

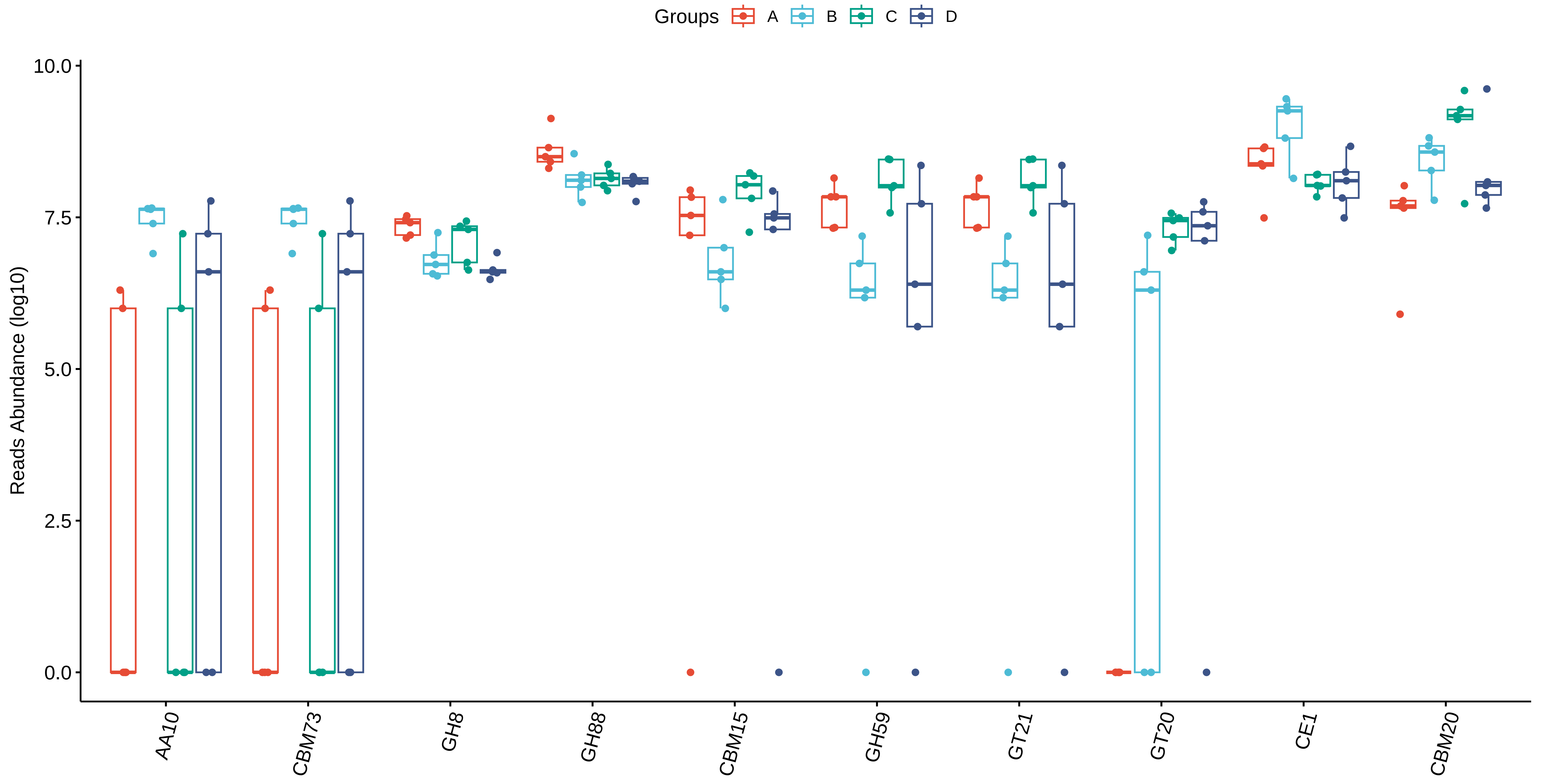

Groups
A
B
C
D
Reads Abundance (log10)
10.0
7.5
5.0
2.5
0.0
AA10
CBM73
GH8
GH88
CBM15
GH59
GT21
GT20
CE1
CBM20
